# Molecular Detection of *Blastocystis* ST2 and ST4 and Exploratory 16S rRNA Gene Community Profiles in a 12-Week Dairy-Intervention Pilot Study

**DOI:** 10.3390/microorganisms14081860

**Published:** 2026-08-21

**Authors:** Panayiota Tsokkou, Chad Schou, Catherine O’Dowd Phanis, Kyriacos A. Hasapis, Stella A. Nicolaou, Eleni P. Andreou, Evelina Charidemou

**Affiliations:** 1 Department of Life Sciences, School of Life and Health Sciences, University of Nicosia, 2417 Nicosia, Cyprus; nutrifun@gmail.com (P.T.); nicolaou.s@unic.ac.cy (S.A.N.); andreou.el@unic.ac.cy (E.P.A.); 2 Department of Basic and Clinical Sciences, University of Nicosia Medical School, 2417 Nicosia, Cyprus; odowd.c@unic.ac.cy (C.O.P.); hasapiskyriacos@gmail.com (K.A.H.)

**Keywords:** *Blastocystis*, 18S rRNA gene, 16S rRNA gene sequencing, gut microbiota, goat milk, dairy-intervention pilot study

## Abstract

*Blastocystis* is a common intestinal protist whose clinical and ecological significance remains uncertain. This secondary exploratory analysis examined *Blastocystis* PCR detection in paired baseline and Week 12 stool samples collected during a randomized pilot intervention comparing daily goat-milk and cow-milk consumption. Nineteen healthy Greek Cypriot adults provided 38 stool samples. Stool DNA was screened by PCR targeting a partial 18S rRNA gene fragment, and positive amplicons were Sanger sequenced for subtype assignment. *Blastocystis* was detected at both time points in 3 of 19 participants (15.8%); ST2 was identified in two participants and ST4 in one. No *Cryptosporidium* spp. or *Giardia duodenalis* PCR-positive samples were identified. In exploratory 16S rRNA gene analyses, median Shannon diversity index was 6.10 versus 4.85 at baseline (*p* = 0.171) and 5.67 versus 4.17 at Week 12 (*p* = 0.085) in repeat-detection and no-detection groups, respectively. Bray–Curtis PERMANOVA was non-significant at baseline (*R*^2^ = 0.0946, *p* = 0.0815) and Week 12 (*R*^2^ = 0.0597, *p* = 0.8576); Aitchison-distance sensitivity analyses were likewise non-significant (*p* = 0.0547 and *p* = 0.3232, respectively). No selected genus-level comparison met the false-discovery-rate-adjusted threshold. The three-participant repeat-detection subgroup precluded reliable analysis of dairy-arm effects, subtype-specific differences, cardiometabolic outcomes, or bacterial-community associations. In this small pilot cohort, ST2 and ST4 were identified in paired baseline and Week 12 stool samples from three healthy adults, providing molecular evidence of repeat detection over the sampled interval. Larger studies with more frequent sampling, quantitative assays, and adequately powered, prespecified analyses are needed to investigate the temporal dynamics and host-associated correlates of *Blastocystis* detection.

## 1. Introduction

The human intestinal microbiome comprises bacteria, archaea, viruses, fungi, and other microeukaryotes [1,2,3]. Although bacterial communities have received substantial attention, the diversity and longitudinal detection of intestinal microeukaryotes remain less well characterized [2,3]. *Blastocystis* is a stramenopile protist frequently detected in human stool and is widely recognized as a common and genetically diverse intestinal protist [4,5,6].

The clinical and ecological significance of *Blastocystis* remains uncertain. It has been detected in both symptomatic and asymptomatic individuals, and its presence alone does not establish either pathogenicity or commensalism [4,6,7,8] [. Observational studies have reported associations between *Blastocystis* detection and higher bacterial diversity, dietary quality, and selected cardiometabolic characteristics [9,10,11,12,13]. However, these studies cannot determine whether *Blastocystis* contributes to these patterns, reflects a pre-existing intestinal environment, or is influenced by other host, dietary, environmental, or microbial factors. Clinical outcomes may also vary according to subtype, organism burden, host susceptibility, co-occurring microorganisms, and the broader intestinal microbial community [4,6,14,15].

Several *Blastocystis* subtypes, including ST2 and ST4, have been reported in humans and non-human hosts [7,16,17,18,19]. This overlap provides a rationale for investigating *Blastocystis* within a One Health framework, particularly in settings where human, livestock, and wildlife habitats intersect. Nevertheless, shared subtypes or sequence similarity across host species cannot independently establish the source, route, direction, or frequency of transmission. Such questions require contemporaneous sampling of humans, animals, and relevant environmental matrices, including water and soil [18,20,21].

The present study used stool samples obtained from healthy Greek Cypriot adults participating in a 12-week randomized pilot trial comparing goat milk and cow milk consumption. Stool samples collected at baseline and Week 12 were screened for *Blastocystis* using 18S rRNA gene PCR and Sanger sequencing. Samples were also screened for *Cryptosporidium* spp. and *Giardia duodenalis* using the specified molecular assays. The 16S rRNA gene sequencing data from the parent study were evaluated as exploratory secondary data.

The aim of this secondary exploratory analysis was to describe *Blastocystis* PCR detection and partial 18S rRNA gene subtype assignment in paired baseline and Week 12 stool samples collected during a randomized dairy-intervention pilot study. Exploratory bacterial 16S rRNA gene, anthropometric, and blood biomarker data were examined descriptively to inform the design of future adequately powered studies.

## 2. Materials and Methods

### 2.1. Study Design, Participants, and Follow-Up

This exploratory analysis used samples collected during a 12-week randomized pilot trial comparing goat milk (GM) and cow milk (CM) consumption in healthy Greek Cypriot adults. This study is a secondary exploratory analysis of samples collected during a randomized dairy-intervention pilot trial. The parent trial enrolled 24 participants aged 19–50 years. Participants were randomly assigned in a 1:1 ratio to GM or CM using a computer-generated blocked randomization sequence with a fixed block size of four. The sequence was generated before enrolment using Randomizer.org by the primary researcher. No stratification factors were used. Allocation was implemented using sequentially numbered, opaque, sealed envelopes prepared according to the randomization sequence. The envelope corresponding to each participant’s enrolment number was opened only after completion of baseline assessments and immediately before provision of milk vouchers.

Participant and investigator blinding were not feasible because goat milk and cow milk differed in sensory characteristics, and the primary researcher delivered the intervention and conducted study visits. Laboratory personnel analyzing blood biomarkers and sequencing personnel responsible for 16S rRNA gene processing were blinded to intervention assignments. Samples submitted for sequencing were relabeled with barcodes that did not reveal intervention assignment.

Participants received vouchers to obtain their assigned commercially available milk from local supermarkets during the 12-week intervention. They were instructed to consume 250 mL of their assigned milk daily for 12 weeks. Adherence was monitored through monthly voucher returns and self-reported milk intake.

Five participants withdrew before the Week 12 assessment because of scheduling conflicts or difficulty adhering to the daily milk-consumption protocol. No withdrawals were attributed to adverse events or gastrointestinal symptoms. The present analysis included the 19 participants who completed the intervention and provided stool samples at both baseline and Week 12. Thus, 38 stool samples were available for molecular screening and 16S rRNA gene community analysis.

Participants reported no antibiotic use during the 12-week study period. Information on regular medications, dietary restrictions, and gastrointestinal symptoms was collected as described in the parent-study protocol.

The primary outcome of this secondary exploratory analysis was *Blastocystis* PCR detection in stool samples collected at baseline and Week 12. 16S rRNA gene community measures and clinical variables were evaluated as secondary exploratory outcomes. The study was not powered to assess milk-type effects among participants with *Blastocystis* detected at both baseline and Week 12, subtype-specific differences, clinical associations, or causal relationships between *Blastocystis* detection and 16S rRNA gene community composition.

### 2.2. Anthropometric and Body Composition Assessment

Anthropometric measurements were obtained at baseline and at Weeks 4, 8, and 12. Height was measured to the nearest centimeter using a portable stadiometer. Body weight and body composition, including fat mass, muscle mass, and total body water, were assessed using a Tanita DC-360 S body-composition monitor (Tanita Corporation, Tokyo, Japan), which uses bioelectrical impedance analysis. Participants were measured barefoot and in light clothing. Body mass index (BMI) was automatically calculated by the device (kg/m^2^). Waist and hip circumferences were measured using a standard tape measure.

### 2.3. Blood Collection and Laboratory Analysis

Fasting biochemical markers were obtained at baseline and Week 12 according to the parent-study protocol. Venous blood samples were collected at baseline and Week 12 following a 12-h overnight fast. Participants were asked to avoid alcohol for three days before blood collection. Samples were collected, transported, and analyzed by a certified clinical laboratory, Bioexelixis, Nicosia, Cyprus under regulated temperature and restricted access protocols.

### 2.4. Stool Collection and DNA Extraction

Stool samples were collected from all participants at baseline and at the end of the study and stored at −80 °C until DNA extraction. To minimize technical variation, all samples were processed in the same laboratory batch. Genomic DNA was extracted from stool using the PureLink™ Microbiome DNA Purification Kit (Invitrogen, Carlsbad, CA, USA) according to the manufacturer’s instructions. Extracted DNA was eluted in nuclease-free buffer and stored at −20 °C until amplification or sent to Novogene GmbH (Munich, Germany) on dry ice for microbiome analysis. All samples were assayed for *Blastocystis* by PCR targeting the small subunit ribosomal RNA gene, and positive amplicons were sequenced. The same extracted DNA aliquot was used for *Blastocystis* PCR screening and 16S rRNA gene sequencing.

### 2.5. Detection of Blastocystis spp.

Stool DNA from 19 participants collected at baseline and Week 12, comprising 38 stool samples, was screened for *Blastocystis* using PCR targeting an approximately 479-bp fragment of the 18S rRNA gene, as described by Santín et al. [22]: Blast 505–532 (forward) 5′-GGAGGTAGTGACAATAAATC-3′ and Blast 998–1017 (reverse) 5′-TGCTTTCGCACTTGTTCATC-3′. Each 25 µL reaction was prepared using the Canvax PCR Kit (Cat No. P0026; Canvax, Córdoba, Spain) and contained: 2.5 µL of 10× PCR Buffer, 2.0 µL of 25 mM MgCl2, 2.5 µL of 8 mM dNTP mix, 1.0 µL (10 pmol) of each primer, 0.25 µL (1.25 U) of Taq Polymerase (5 U/µL), 11.75 µL of sterile dH2O, and 4 µL of template DNA. The cycling conditions consisted of an initial denaturation at 95 °C for 5 min, followed by 35 cycles of 95 °C for 30 s, 54 °C for 30 s, and 72 °C for 60 s, with a final extension at 72 °C for 5 min. PCR products were electrophoresed on 1% agarose gels, stained with GelRed Prestain Plus 6× DNA Loading Dye (Biotium, Fremont, CA, USA), and visualized under ECX-F20.M-V1 imaging system (Vilber Lourmat, Collégien, France). Each PCR run included a known *Blastocystis* ST4-positive control and a nuclease-free-water negative control.

### 2.6. Detection of Cryptosporidium spp. and Giardia Duodenalis

All 38 samples were also screened for *Cryptosporidium* spp. and *Giardia duodenalis* using nested-PCR assays targeting the 18S rRNA gene and triosephosphate isomerase (tpi) gene, respectively.

*Cryptosporidium* spp. screening was conducted using the nested-PCR protocol of Silva et al. [23]. The primary PCR used primers SHP1 (5′-ACC TAT CAG CTT TAG ACG GTA GGG TAT-3′) and SHP2 (5′-TTC TCA TAA GGT GCT GAA GGA GTA AGG-3′), and the secondary nested reaction used primers SHP3 (5′-ACA GGG AGG TAG TGA CAA GAA ATA ACA-3′) and SSU-R3 (5′-AAG GAG TAA GGA ACA ACC TCC A-3′). Both PCR rounds included an initial denaturation at 94 °C for 3 min; 35 cycles of 94 °C for 45 s, 56 °C for 45 s, and 72 °C for 60 s; followed by a final extension at 72 °C for 7 min.

*Giardia duodenalis* screening was conducted using the nested-PCR protocol described by Sulaiman et al. [24]. The primary reaction used primers AL3543 (5′-AAATIATGCCTGCTCGTCG-3′) and AL3546 (5′-CAAACCTTITCCGCAAACC-3′), and the nested reaction used internal primers AL3544 (5′-CCCTTCATCGGIGGTAACTT-3′) and AL3545 (5′-GTGGCACCACICCCGTGCC-3′). Both reactions included an initial denaturation at 95 °C for 5 min; 35 cycles of denaturation at 94 °C for 60 s, annealing at 54 °C for the primary reaction or 58 °C for the nested reaction for 60 s, and extension at 72 °C for 60 s; followed by a final extension at 72 °C for 10 min.

PCR products were assessed by 1% agarose-gel electrophoresis with appropriate molecular-weight markers. Positive and negative controls were included in each run. No amplicons were detected for *Cryptosporidium* spp. or *G. duodenalis* using these assays. This screening was limited to these two protozoan taxa and did not constitute comprehensive profiling of intestinal eukaryotes or parasites.

### 2.7. Sequencing and Blastocystis Subtype Assignment

PCR products of the expected size were excised from agarose gels and purified using the ExtractMe DNA Gel-Out Kit (Cat. No. EM26.1-050; Blirt, Gdańsk, Poland). Purified amplicons were submitted to Macrogen Europe (Amsterdam, The Netherlands) for Sanger sequencing using the forward PCR primer.

Chromatograms were visually inspected and trimmed before sequence analysis. The resulting sequences were queried against the National Center for Biotechnology Information nucleotide database using BLAST (version Blast+2.17.0) to confirm *Blastocystis* identity and identify closely related reference sequences. Multiple-sequence alignments were generated using MUSCLE in MEGA version 11 [25].

Subtype assignments were supported by BLAST sequence similarity and phylogenetic clustering with subtype reference sequences. Study sequences were deposited in GenBank under accession numbers PX776016–PX776021.

### 2.8. Phylogenetic Analysis

Phylogenetic analysis was conducted in MEGA version 11 [25]. Study sequences and selected *Blastocystis* reference sequences retrieved from GenBank were aligned using MUSCLE. A neighbor-joining tree was inferred using the Kimura two-parameter model and 1000 bootstrap replicates. *Proteromonas lacertae* (GenBank accession U37108) was used as the outgroup.

The phylogenetic analysis was used to support subtype assignment. Comparisons with publicly available sequences from non-human hosts were interpreted as sequence-similarity observations only and were not used to infer transmission source, direction, or zoonotic transmission.

### 2.9. Bacterial 16S rRNA Gene Sequencing and Bioinformatic Processing

The 16S rRNA gene community profiling was performed by Novogene GmbH (Munich, Germany). The V3–V4 hypervariable region of the 16S rRNA gene was amplified using the forward primer 5′-CCTAYGGGRBGCASCAG-3′ and reverse primer 5′-GGACTACNNGGGTATCTAAT-3′. Sequencing libraries were prepared using the NEBNext Ultra II DNA Library Prep Kit (New England Biolabs, Ipswich, MA, USA) and sequenced on the Illumina NovaSeq 6000 platform (Illumina, San Diego, CA, USA) using 250-bp paired-end reads.

Bioinformatic processing was performed by Novogene using QIIME 2 version 2022.02. Primer sequences and short reads were removed using Cutadapt version 3.3. Paired-end reads were merged using FLASH version 1.2.11. Tags were quality filtered using fastp version 0.23.1. Denoising and amplicon sequence variant inference were conducted using DADA2 within QIIME 2, and chimeric sequences were removed using VSEARCH version 2.16.0. Taxonomy was assigned using the QIIME 2 classify-sklearn method with a pre-trained Naive Bayes classifier against the SILVA version 138.2 reference database.

The 16S rRNA gene dataset comprised 38 stool samples collected from 19 participants at baseline and Week 12. The non-rarefied ASV feature table contained 22,455–56,490 processed sequencing reads per sample. For rarefaction-based alpha-diversity analyses, all samples were rarefied to 22,455 reads per sample, corresponding to the minimum sequencing depth across the 38 samples. This threshold retained all samples. Alpha-diversity metrics, including the Shannon index, Chao1 richness, observed features, and Pielou’s evenness, were calculated from the rarefied dataset. Rarefaction was used to address uneven sequencing depth in amplicon-sequencing analyses [26]. Sample-level processed sequencing-read counts before and after rarefaction are reported in Supplementary Table S3, and the sequencing-depth distribution is shown in Supplementary Figure S1.

The 16S rRNA gene data were used to evaluate exploratory community-composition patterns. Because 16S rRNA gene sequencing provides taxonomic rather than direct functional information, no conclusions regarding microbial metabolic pathways, short-chain fatty acid production, inflammatory activity, or other functional capacities were drawn from these data.

### 2.10. Taxonomic Aggregation and Data Availability

The sequencing facility provided an ASV feature table. ASV-level data were retained for sequence-quality assessment and rarefaction-based alpha-diversity analyses. For selected exploratory taxonomic summaries, Bray–Curtis community analyses, and the compositional sensitivity analysis, ASVs were aggregated to genus-level taxonomic count data by summing reads assigned to the same genus. The Novogene Others category was retained for community-level analyses to preserve sample-level processed-count totals.

Genus-level aggregation was used to reduce feature dimensionality and facilitate interpretation of exploratory taxonomic summaries. It was not intended to increase biological resolution or establish species-level or strain-level differences. Accordingly, genus-level aggregation may obscure species-level, strain-level, or ASV-level variation. For rarefied genus-level feature tables, zero values indicate that no reads assigned to the taxon were observed after the stated processing and rarefaction workflow; they do not demonstrate biological absence.

Raw sequencing reads are available in the National Center for Biotechnology Information Sequence Read Archive under BioProject accession number PRJNA1464099. The Supplementary Materials includes processed ASV and genus-level data, sample metadata, and sequencing-quality information.

### 2.11. Statistical Analysis

Statistical analyses were performed using GraphPad Prism version 10.0 (GraphPad Software, Boston, MA, USA), R version 4.6.0 with the vegan package version 2.7–5, and Python version 3.12.13 with SciPy version 1.16.3 and statsmodels version 0.14.6. Participant-level demographic data were summarized as means and standard deviations or medians and interquartile ranges, as appropriate. Given the small number of participants with *Blastocystis* detected at both baseline and Week 12 (*n* = 3), comparisons according to *Blastocystis* detection status were considered exploratory.

*Blastocystis* PCR detection at baseline and Week 12, together with subtype distribution, was summarized descriptively at the participant level. No formal comparison between ST2 and ST4 was performed because ST2 was identified in two participants and ST4 in one participant. Similarly, no inferential comparison of goat milk and cow milk effects was undertaken among participants with *Blastocystis* detected at both time points.

Anthropometric and biochemical variables were summarized descriptively according to *Blastocystis* PCR-detection status at baseline and Week 12. Given the three-participant repeat-detection subgroup, these data were not used for inferential assessment of clinical, metabolic, or anthropometric differences.

The eight genera presented in the exploratory taxonomic analysis were selected because they have been discussed in prior literature concerning *Blastocystis*, dietary exposures, or gut microbial ecology. These analyses were exploratory, and the selected genera were not treated as confirmatory endpoints.

Community composition was evaluated separately at baseline and Week 12 to avoid treating repeated samples from the same participant as independent observations. Bray–Curtis dissimilarities were calculated from genus-level relative abundances derived from non-rarefied processed 16S rRNA gene count data. Permutational multivariate analysis of variance was performed using the adonis2 function in the vegan package with 9999 permutations. *Blastocystis* detection status was included as the explanatory variable, and effect sizes were reported as *R*^2^ values.

Homogeneity of multivariate dispersion was assessed separately at each time point using PERMDISP, implemented with the betadisper function in vegan and evaluated using 9999 permutations. A non-significant PERMDISP result was interpreted as no detected evidence of differential dispersion under the specified analysis, rather than confirmation of equal dispersion.

As a compositional sensitivity analysis, non-rarefied genus-level count data were analyzed separately at baseline and Week 12. A pseudocount of 1 was added to zero counts before centered log-ratio transformation. Euclidean distances calculated from centered log-ratio-transformed data were treated as Aitchison distances. PERMANOVA was then performed using 9999 permutations.

Selected genus-level comparisons were conducted as exploratory analyses using relative abundances calculated from genus-level counts aggregated from the ASV feature table after rarefaction to 22,455 reads per sample. Two-sided Mann–Whitney U tests were performed separately at baseline and Week 12 using SciPy. Unadjusted *p*-values were adjusted within each time point across the eight selected genera using the Benjamini–Hochberg false-discovery-rate procedure implemented in statsmodels. Findings with *q* ≥ 0.05 were not interpreted as statistically significant.

Mann–Whitney U tests were two-sided. A nominal threshold of *p* < 0.05 was used for exploratory interpretation, and genus-level analyses were additionally evaluated after Benjamini–Hochberg false-discovery-rate adjustment. Given the pilot sample size and limited number of participants with *Blastocystis* detection, statistical estimates were interpreted cautiously.

### 2.12. Ethics Approval

The study was conducted in accordance with the Declaration of Helsinki. The study protocol was approved by the Cyprus National Bioethics Committee, approval number EEBK/EΠ/2023/22. All participants provided written informed consent before enrollment and sample collection.

## 3. Results

### 3.1. Participant Completion and Blastocystis Detection

Of the 24 participants enrolled in the parent dairy-intervention pilot trial, 19 completed the 12-week intervention and provided stool samples at baseline and Week 12 (Table 1). The final analytical dataset therefore comprised 38 stool samples from 19 participants. Nine participants were assigned to the goat milk group and 10 to the cow milk group. Among the 19 completers, *Blastocystis* was detected by PCR in three participants at baseline and again in the same three participants at Week 12, corresponding to a point prevalence of 15.8% at each sampled time point. The three participants with repeat *Blastocystis* PCR detection were designated G10, G16, and C23. Participant G10 was assigned to the goat-milk group and carried ST4. Participants G16 and C23 carried ST2 and were assigned to the goat-milk and cow-milk groups, respectively. The remaining 16 participants had no *Blastocystis* PCR detection at either baseline or Week 12.

All 38 stool samples were negative for *Cryptosporidium* spp. and *Giardia duodenalis* by the specified nested-PCR assays. No gastrointestinal symptoms were reported by the three participants with *Blastocystis* detection during the intervention period.

### 3.2. Molecular Characterization and Phylogenetic Analysis

Sequencing of PCR-positive amplicons identified *Blastocystis* ST2 in two participants and ST4 in one participant. Partial 18S rRNA gene sequences obtained from each positive participant at baseline and Week 12 were assigned to the same subtype at both sampling time points. The baseline and Week 12 sequences were identical within each participant across the analyzed partial 18S rRNA gene region.

**Figure 1:**
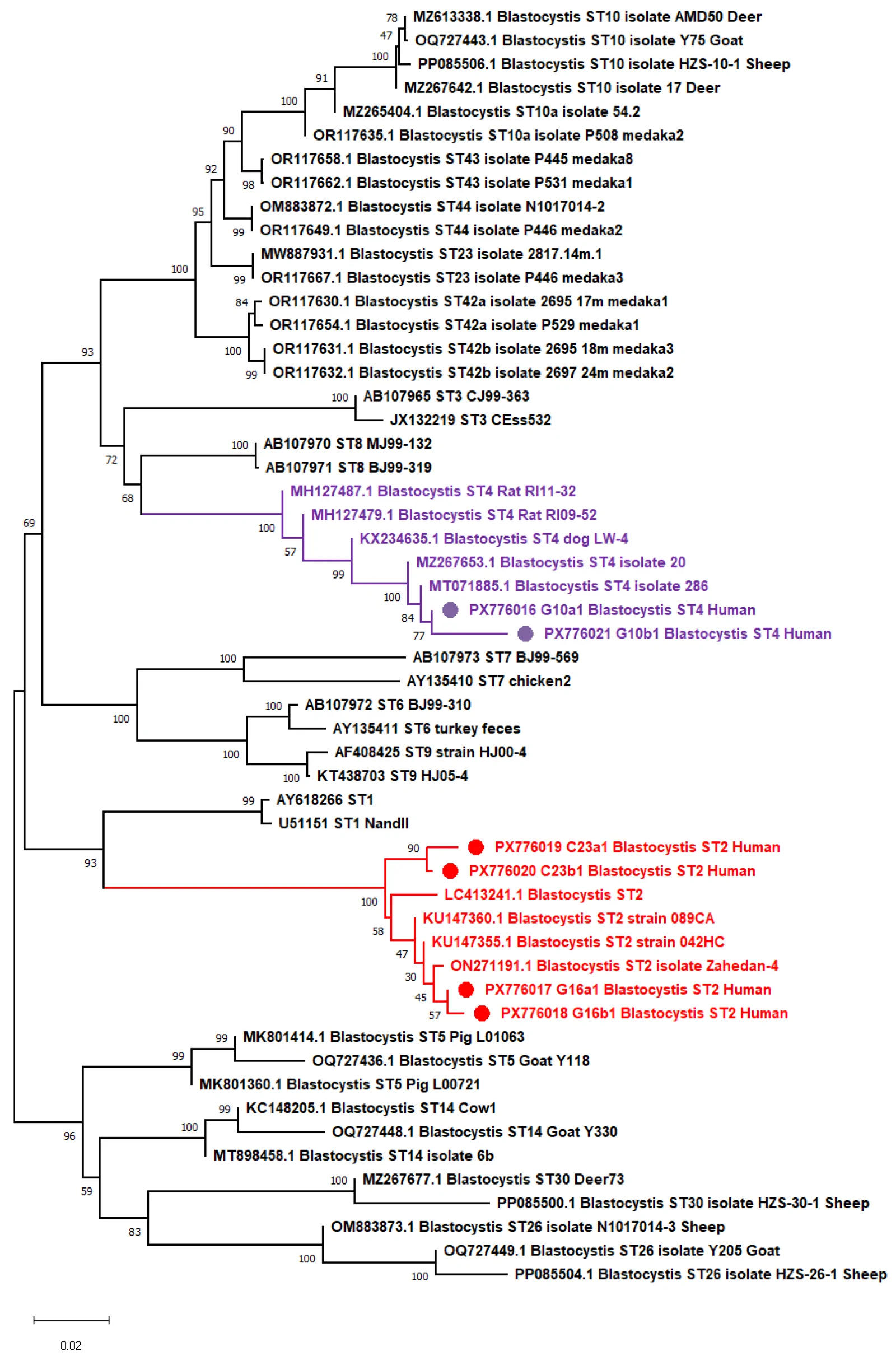
Phylogenetic Analysis of *Blastocystis* Partial 18S rRNA Gene Sequences. Neighbor-joining phylogenetic tree based on partial 18S rRNA gene sequences obtained from *Blastocystis*-positive stool samples. Evolutionary distances were calculated using the Kimura two-parameter model, and branch support was assessed using 1000 bootstrap replicates. Study sequences are indicated by filled circles and cluster with ST2 or ST4 reference sequences. *Proteromonas lacertae* (GenBank accession U37108) was used as the outgroup. Bootstrap values are shown above branches where applicable. Filled circles indicate sequences generated in the present study. Purple and red labels identify study sequences assigned to ST4 and ST2, respectively.

Phylogenetic analysis supported clustering of the study sequences with ST2 and ST4 reference sequences, respectively (Figure 1, Table 2). The six study sequences were deposited in GenBank under accession numbers PX776016–PX776021. Because animal, wildlife, water, and other environmental samples were not collected in the present study, no inference regarding zoonotic transmission or transmission pathways was made.

### 3.3. Exploratory 16S rRNA Gene Alpha- and Beta-Diversity Analyses

Exploratory alpha-diversity and selected bacterial-genus comparisons, together with descriptive anthropometric and blood-biomarker summaries, were limited by the three-person repeat-detection subgroup. No selected genus-level comparison met the false-discovery-rate-adjusted threshold. Alpha-diversity and taxonomic results, together with descriptive anthropometric and biochemical summaries, are provided in the Supplementary Materials for transparency and hypothesis generation.

16S rRNA gene sequencing was completed for all 38 stool samples. Following quality control, all samples were rarefied to 22,455 reads per sample for rarefaction-based alpha-diversity. At baseline, the three participants with *Blastocystis* detected at both time points had a median Shannon diversity index of 6.10 [IQR: 4.67–6.24], compared with 4.85 [IQR: 4.27–5.13] among participants without *Blastocystis* detection (Table 3). At Week 12, the corresponding median Shannon index values were 5.67 [IQR: 4.53–6.22] and 4.17 [IQR: 3.54–5.38], respectively. Exploratory Mann–Whitney U tests did not identify statistically significant between-group differences in Shannon diversity at baseline or Week 12.

Similar descriptive patterns were observed for Pielou’s evenness, Chao1 richness, and observed features. However, these comparisons were based on only three participants with *Blastocystis* detected at both sampling time points and were not interpreted as confirmatory evidence of a *Blastocystis*-associated 16S rRNA gene diversity profile.

16S rRNA gene community composition was evaluated separately at baseline and Week 12 using Bray–Curtis dissimilarities calculated from genus-level relative abundances derived from non-rarefied processed count data. At baseline, *Blastocystis* detection status accounted for 9.5% of variation in community composition (*R*^2^ = 0.0946, pseudo-*F* = 1.778, *p* = 0.0815; Table 4). At Week 12, *Blastocystis* detection status accounted for 6.0% of variation in community composition (*R*^2^ = 0.0597, pseudo-*F* = 1.078, *p* = 0.8576). Neither Bray–Curtis analysis produced a permutation *p*-value below 0.05. PERMDISP did not identify statistically detectable differences in multivariate dispersion between detection groups at baseline (*p* = 0.8531) or Week 12 (*p* = 0.1517; Supplementary Table S4). Given the small and unbalanced groups, these findings do not establish equal dispersion and were interpreted cautiously.

Given the three-participant repeat-detection subgroup, unequal group sizes, and paired sampling structure, these analyses were considered exploratory and were not interpreted as evidence of a group-level community difference. In the compositional sensitivity analysis using centered log-ratio-transformed non-rarefied genus-level count data and Aitchison distance, PERMANOVA results were also non-significant at baseline (*R*^2^ = 0.0848, pseudo-*F* = 1.5755, *p* = 0.0547) and Week 12 (*R*^2^ = 0.0582, pseudo-*F* = 1.0609, *p* = 0.3232; Table 4). Accordingly, beta-diversity analyses were interpreted as exploratory and insufficient to establish a distinct 16S rRNA gene community profile associated with *Blastocystis* detection.

### 3.4. Exploratory Taxonomic Comparisons

Exploratory genus-level comparisons were conducted separately at baseline and Week 12 using relative abundances obtained from the Novogene genus-level table derived from ASV data rarefied to 22,455 reads per sample. At baseline, *Alistipes* had an unadjusted *p*-value below 0.05 (*p* = 0.0475). No other selected genus had an unadjusted *p*-value below 0.05 at baseline or Week 12. After Benjamini–Hochberg false-discovery-rate adjustment, no genus-level comparison met the applied threshold of *q* < 0.05 at either time point. The smallest adjusted value was *q* = 0.3385 at baseline. The estimated taxonomic differences were imprecise, and no selected genus remained associated with *Blastocystis* PCR detection after false-discovery-rate adjustment.

Full relative-abundance summaries, unadjusted *p*-values, FDR-adjusted *q*-values, and details on zero values are provided in Supplementary Table S5. Zero values indicate that no reads assigned to the taxon were observed after the stated processing and rarefaction workflow; they do not demonstrate biological absence.

### 3.5. Exploratory Clinical and Anthropometric Data

Anthropometric and biochemical measurements were summarized descriptively according to *Blastocystis* PCR-detection status and are presented in Supplementary Table S1. Individual baseline and Week 12 measurements for the three participants with *Blastocystis* detected at both time points are provided in Supplementary Table S2 for transparency. These data were not interpreted as evidence of treatment effects, metabolic benefit, clinical differences, or subtype-specific effects.

## 4. Discussion

### 4.1. Principal Findings

In this exploratory analysis of a 12-week randomized dairy-intervention pilot study, *Blastocystis* was detected by PCR in 3 of 19 healthy Greek Cypriot adults, corresponding to a participant-level prevalence of 15.8%. Partial 18S rRNA gene sequencing identified ST2 in two participants and ST4 in one participant. The same subtype was identified in paired baseline and Week 12 samples from each participant with *Blastocystis* PCR detection. All three participants with *Blastocystis* detection reported no gastrointestinal symptoms during the study period, and no *Cryptosporidium* spp. or *Giardia duodenalis* amplicons were detected using the specified nested-PCR assays. The study illustrates the feasibility of integrating paired molecular screening, partial *Blastocystis* subtype characterization, and bacterial 16S rRNA gene profiling within a structured randomized dietary-intervention setting.

The small number of participants with *Blastocystis* detection limits interpretation. In particular, the present study was not powered to determine whether goat milk or cow milk influenced *Blastocystis* detection, whether ST2 and ST4 differed in their ecological or clinical associations, or whether *Blastocystis* detection was associated with 16S rRNA gene community composition or cardiometabolic outcomes. The findings should therefore be interpreted as descriptive pilot data that may inform the design of larger prospective studies.

### 4.2. Blastocystis Detection at Baseline and Week 12

The detection of ST2 and ST4 in this healthy adult cohort is consistent with reports that these subtypes occur commonly in human populations [4,5,6,7]. The clinical significance of *Blastocystis* spp. remains uncertain because it has been identified in both symptomatic and asymptomatic individuals [4,6,7,8]. Accordingly, the absence of reported gastrointestinal symptoms among the three participants with repeat *Blastocystis* PCR detection should not be interpreted as evidence that ST2 or ST4 is universally non-pathogenic, nor does it establish commensalism in this cohort.

The same subtype was identified at baseline and Week 12 in all three participants with repeat *Blastocystis* PCR detection. This observation is consistent with repeat detection over the sampled interval. However, the study included only two stool-collection time points and did not quantify *Blastocystis* burden. Therefore, it cannot establish uninterrupted carriage, acquisition or clearance between visits, or the duration of colonization before enrolment. Intermittent shedding and variation in organism burden may also affect molecular detection in stool samples [6,10].

Experimental and observational evidence suggests that the effects associated with *Blastocystis* may depend on subtype, parasite factors, host susceptibility, and the surrounding microbial environment [4,6,14,15]. For example, pathogenic mechanisms have been described for ST7 in experimental studies [14,15], whereas experimental colonization studies of ST1 and ST4 have reported microbiota and immune effects in mouse colitis models [27,28]. These experimental observations should not be extrapolated to infer the clinical role of ST2 or ST4 in the present healthy adult cohort.

### 4.3. Regional and One Health Context

The 15.8% prevalence observed at each sampled time point may be compared descriptively with regional reports, including a prevalence of 27.8% reported in a North Cyprus study [29]. Differences between studies may reflect population characteristics, recruitment setting, clinical status, sample size, diagnostic method, environmental exposures, and subtype distribution. The present study was not designed to estimate population prevalence in Cyprus.

ST2 and ST4 have been reported in humans and non-human hosts, including livestock and other animals [7,16,17,18,19]. However, this study did not include contemporaneous sampling of livestock, wildlife, food, water, soil, or other environmental matrices. It therefore cannot identify infection sources or infer zoonotic, environmental, or waterborne transmission. Integrated human–animal–environmental sampling with higher-resolution molecular approaches and exposure data would be required to investigate these questions [18,20,21].

### 4.4. Exploratory 16S rRNA Gene Community Findings

The repeat-detection subgroup had higher median values for Shannon diversity, Pielou’s evenness, Chao1 richness, and observed features at the reported time points. However, none of these exploratory comparisons reached statistical significance, and the estimates were based on only three participants with repeat *Blastocystis* PCR detection. Previous observational studies have reported associations between *Blastocystis* detection and higher bacterial diversity in human cohorts [9,10,11,12,13]. The present results are broadly consistent with that literature at a descriptive level, but they do not establish a reproducible diversity pattern associated with *Blastocystis* detection.

The beta-diversity findings require particular caution. Neither the Bray–Curtis nor Aitchison PERMANOVA analyses met the applied statistical significance threshold at baseline or Week 12. At baseline, the estimated effect sizes were modest, but the small number of participants with *Blastocystis* detection and the non-significant permutation results preclude inference of a distinct 16S rRNA gene community-composition profile.

In exploratory genus-level comparisons, *Alistipes* had an unadjusted *p*-value below 0.05 at baseline. However, no selected genus met the applied false-discovery-rate-adjusted threshold at baseline or Week 12. These taxonomic findings should not be interpreted as evidence of enrichment or depletion of specific genera according to *Blastocystis* detection status. The observed microbiota patterns were heterogeneous across the three participants with *Blastocystis* detected at both time points, and the study cannot define a common *Blastocystis*-associated 16S rRNA gene community profile.

The 16S rRNA gene sequencing approach provides taxonomic information but does not directly measure microbial genes, transcripts, metabolites, short-chain fatty acids, inflammatory pathways, or host immune responses. Therefore, the present study cannot make functional inferences regarding butyrate production, mucosal integrity, lipopolysaccharide biosynthesis, inflammatory activity, or other metabolic pathways. Future studies should combine repeated *Blastocystis* measurement with shotgun metagenomics, metabolomics, and relevant host biomarkers.

### 4.5. Dairy-Intervention Context and Clinical Measurements

The randomized dairy-intervention setting provided paired stool samples from healthy adults under a structured dietary protocol. However, only three participants had *Blastocystis* PCR detection at baseline and Week 12, including two allocated to goat milk and one allocated to cow milk. This distribution precluded meaningful analysis of milk-type effects on *Blastocystis* detection, bacterial community composition, or clinical variables.

Existing studies relating diet to *Blastocystis* have mainly examined overall dietary patterns rather than controlled goat-milk versus cow-milk interventions [9,10,11]. Although goat milk and cow milk differ in nutritional composition [30,31,32,33], this pilot dataset cannot determine whether either milk type influenced *Blastocystis* detection. Anthropometric and biochemical data are therefore retained as exploratory descriptive results only.

### 4.6. Strengths and Limitations

This study provides a paired molecular dataset from a randomized dietary-intervention setting, combining *Blastocystis* PCR screening, partial 18S rRNA gene sequencing and subtype assignment, screening for *Cryptosporidium* spp. and *Giardia duodenalis*, and bacterial 16S rRNA gene profiling from the same stool-DNA preparations. Complete paired sampling of 19 intervention completers, deposition of *Blastocystis* sequences in GenBank, and deposition of 16S rRNA gene reads in the Sequence Read Archive support transparency and future comparative research.

The principal limitation is the small number of participants with *Blastocystis* detected at both baseline and Week 12 (*n* = 3). This limited statistical power prevented reliable subtype-specific analysis and precluded testing of milk-type effects among these participants. The study also included only two stool-sampling time points, did not quantify *Blastocystis* burden, and did not comprehensively characterize other intestinal eukaryotes, fungi, viruses, or parasites. Although *Cryptosporidium* spp. and *G. duodenalis* were not detected, this does not exclude other co-occurring intestinal organisms.

Participant and investigator blinding were not feasible because of sensory differences between goat milk and cow milk. In addition, the primary researcher was involved in several trial procedures, including randomization, participant enrolment, intervention delivery, and study visits. This may have introduced performance or detection bias despite blinded laboratory and sequencing analyses.

Additional limitations include the absence of direct functional measurements, such as metagenomic, metatranscriptomic, metabolomic, immune, or inflammatory analyses. The lack of animal, wildlife, water, soil, and food sampling also prevents inference regarding zoonotic or environmental transmission. Finally, beta-diversity and taxonomic analyses were exploratory. Neither Bray–Curtis nor Aitchison PERMANOVA met the applied statistical significance threshold, reinforcing the need for cautious interpretation. Despite these limitations, the paired sample collection, subtype-resolved molecular detection, matched bacterial 16S rRNA gene data, and public deposition of sequence data provide a reproducible foundation for larger studies designed to test specific hypotheses regarding *Blastocystis* detection, subtype distribution, diet, and gut microbial ecology.

Larger prospective studies should include more frequent stool sampling, quantitative *Blastocystis* molecular assays, comprehensive multi-kingdom microbiome profiling, assessment of dietary exposure and adherence, functional multi-omics data, and integrated human–animal–environmental sampling. Such studies would be better positioned to assess the determinants and clinical relevance of *Blastocystis* detection in healthy and symptomatic populations.

## 5. Conclusions

In this secondary exploratory analysis of a 12-week randomized dairy-intervention pilot study, *Blastocystis* was detected by PCR at baseline and Week 12 in three of 19 healthy Greek Cypriot adults. Partial 18S rRNA gene sequencing assigned two participants to ST2 and one participant to ST4. The same subtype was identified in paired samples from each participant, providing evidence of repeat detection at the two sampled time points.

The three-participant repeat-detection subgroup precluded reliable inference regarding goat-milk versus cow-milk effects, subtype-specific differences, cardiometabolic outcomes, or bacterial 16S rRNA gene community profiles. Alpha-diversity, beta-diversity, and selected taxonomic comparisons were exploratory and did not provide statistically significant evidence of a distinct *Blastocystis*-associated bacterial community profile. The study cannot establish uninterrupted carriage, commensal status, functional microbiome effects, causal milk-type effects on *Blastocystis* detection, or transmission pathways. Larger prospective studies with repeated sampling, quantitative molecular assays, comprehensive multi-kingdom profiling, functional measurements, and appropriately powered prespecified analyses are needed.

## Figures and Tables

**Table 1:** Characteristics of Participants According to *Blastocystis* PCR Detection Status.

Characteristic	*Blastocystis* Detected at Baseline and Week 12, *n* = 3	*Blastocystis* Not Detected at Baseline and Week 12, *n* = 16
Age, years, median [IQR]	40.0 [31.0–48.0]	41.0 [38.3–44.8]
Sex, male/female	1/2	2/14
Randomized allocation, goat milk/cow milk	2/1	7/9
Baseline BMI, kg/m^2^, median [IQR]	22.93 [22.4–24.5]	23.0 [20.7–24.7]
Week 12 BMI, kg/m^2^, median [IQR]	21.6 [21.5–24.2]	22.8 [20.3–24.7]

Note: This table describes the analytical cohort. Given the small number of participants with *Blastocystis* detection, these data should not be used to infer group equivalence or absence of confounding. See Supplementary Table S1 for the complete demographic, anthropometric, and biochemical dataset.

**Table 2:** Participant-Level *Blastocystis* Detection and Subtype Assignment.

Participant	Dairy-Intervention Arm	Baseline Result	Week 12 Result	*Blastocystis* Subtype (ST)	Baseline GenBank Accession	Week 12 GenBank Accession
G10	Goat milk	Detected	Detected	ST4	PX776016	PX776021
G16	Goat milk	Detected	Detected	ST2	PX776017	PX776018
C23	Cow milk	Detected	Detected	ST2	PX776019	PX776020

Note: Subtypes were assigned using partial 18S rRNA gene PCR, Sanger sequencing, BLAST sequence comparison, and phylogenetic clustering with reference sequences. “Detected” refers to PCR detection in the analyzed baseline or Week 12 stool sample and does not demonstrate uninterrupted carriage between the two sampling time points.

**Table 3:** Exploratory Alpha-Diversity Measures by *Blastocystis* Detection Status.

Measure	Time Point	*Blastocystis* Detected, *n* = 3, Median [IQR]	*Blastocystis* Not Detected, *n* = 16, Median [IQR]	Mann–Whitney U *p*-Value
Shannon index	Baseline	6.10 [4.67–6.24]	4.85 [4.27–5.13]	0.171
	Week 12	5.67 [4.53–6.22]	4.17 [3.54–5.38]	0.085
Pielou’s evenness	Baseline	0.75 [0.64–0.75]	0.66 [0.59–0.68]	0.171
	Week 12	0.72 [0.65–0.74]	0.59 [0.52–0.68]	0.171
Chao1 richness	Baseline	306.5 [168.7–322.7]	177.6 [160.9–220.8]	0.171
	Week 12	344.5 [174.1–345.2]	184.1 [122.2–265.7]	0.109
Observed features	Baseline	291.0 [163.0–317.0]	168.5 [150.8–201.0]	0.105
	Week 12	327.0 [153.0–335.0]	162.5 [119.8–253.3]	0.138

Note: Alpha-diversity measures were calculated from ASV data rarefied to 22,455 reads per sample. Tests were exploratory and should not be interpreted as confirmatory evidence of between-group differences.

**Table 4:** Exploratory PERMANOVA Analyses of 16S rRNA Gene Community Composition by *Blastocystis* Detection Status.

Distance Metric and Time Point	Factor	*df*	Sum of Squares	Pseudo-*F*	*R* ^2^	Permutation *p*-Value
Bray–Curtis, baseline	*Blastocystis* detection status	1	0.302	1.778	0.0946	0.0815
Bray–Curtis, Week 12	*Blastocystis* detection status	1	0.297	1.078	0.0597	0.8576
Aitchison, baseline	*Blastocystis* detection status	1	639.688	1.576	0.0848	0.0547
Aitchison, Week 12	*Blastocystis* detection status	1	594.889	1.061	0.0582	0.3232

Note: Bray–Curtis dissimilarities were calculated from genus-level relative abundances derived from non-rarefied processed 16S rRNA gene count data. Aitchison distances were calculated as Euclidean distances from centered log-ratio-transformed non-rarefied genus-level count data after addition of a pseudocount of 1. PERMANOVA analyses were performed separately at baseline and Week 12 using 9999 permutations. PERMDISP analyses of multivariate dispersion are reported in Supplementary Table S4. Analyses were exploratory because only three participants had *Blastocystis* detected at both sampling time points.

## Data Availability

Raw 16S rRNA gene sequencing reads generated during this study are available in the National Center for Biotechnology Information Sequence Read Archive under BioProject accession number PRJNA1464099. The study is associated with Novogene Project ID X204SC25095874-Z01. Partial *Blastocystis* 18S rRNA gene sequences have been deposited in GenBank under accession numbers PX776016–PX776021. Supporting processed count tables, sample metadata, clinical data, and supplementary analyses are provided in the article, Supplementary Materials, and accompanying Supplementary File S1.

## Supplementary Materials

The following supporting information can be downloaded at: https://www.mdpi.com/article/10.3390/microorganisms14081860/s1, Supplementary Table S1: Exploratory descriptive demographic, anthropometric, and biochemical summaries according to *Blastocystis* PCR-detection status; Supplementary Table S2: Descriptive anthropometric and HOMA-IR measurements for participants with *Blastocystis* detected at baseline and week 12; Supplementary Table S3: Processed sequencing reads before and after rarefaction; Supplementary Table S4: PERMDISP analysis of bray-curtis dissimilarities by *Blastocystis* detection status; Supplementary Table S5: Exploratory relative-abundance comparisons for eight selected bacterial genera according to *Blastocystis* PCR-detection status; Supplementary Figure S1: Processed sequencing depth across the 38 stool samples; Supplementary File S1: Blastocystis Diary Pilot Study Supplementary Data.
