# Supplementary material for: Molecular Detection of Blastocystis ST2 and ST4 and Exploratory 16S rRNA Gene Community Profiles in a 12-Week Dairy-Intervention Pilot Study
Source: Microorganisms. 2026 Aug 21;14(8):1860. doi: 10.3390/microorganisms14081860 (PMC13515294; doi:10.3390/microorganisms14081860)
Supplement: Supplementary file 1 [file microorganisms-14-01860-s001.zip › microorganisms-4447473-supplementary.pdf]

Supplementary Table S1. Exploratory Descriptive Demographic, Anthropometric, and Biochemical Summaries According to *Blastocystis* PCR-Detection Status

Category	Parameter	Visit	*Blastocystis* detected at baseline and Week 12, *n* = 3	*Blastocystis* not detected at baseline and Week 12, *n* = 16
Demographics	Age (years)	Baseline	40.0 [31.0–48.0]	41.0 [38.3–44.8]
	Sex (M/F)	Baseline	1 / 2	2 / 14
Anthropometric	BMI (kg/m²)	Baseline	22.93 [22.4–24.5]	23.0 [20.7–24.7]
		Week 12	21.6 [21.5–24.2]	22.8 [20.3–24.7]
	Body fat %	Baseline	27.5 [14.5–28.7]	27.4 [21.0–32.2]
		Week 12	23.5 [13.1–28.6]	25.5 [17.9–31.9]
	Waist-to-Hip Ratio	Baseline	0.80 [0.75–0.83]	0.82 [0.80–0.89]
		Week 12	0.81 [0.71–0.82]	0.80 [0.75–0.89]
Blood Chemistry	Fasting Glucose (mg/dL)	Baseline	86.3 [79.4–90.6]	91.2 [85.7–98.8]
		Week 12	78.6 [76.8–87.9]	82.8 [76.3–88.7]
	Fasting Insulin (μIU/mL)	Baseline	4.7 [2.3–5.7]	7.6 [4.9–9.4]
		Week 12	2.9 [2.0–9.5]	6.7 [5.7–8.0]
	HOMA-IR	Baseline	1.1 [0.5–1.1]	1.6 [1.1–2.2]
		Week 12	0.5 [0.4–2.1]	1.4 [1.1–1.6]
	Triglycerides (mg/dL)	Baseline	60.0 [38.1–71.6]	76.3 [55.0–110.9]
		Week 12	60.7 [46.0–62.4]	82.7 [58.9–111.3]
	HDL- cholesterol (mg/dL)	Baseline	74.1 [63.1–87.7]	76.2 [65.9–88.6]
		Week 12	59.5 [36.6–68.8]	62.9 [54.9–74.5]
	LDL- cholesterol (mg/dL)	Baseline	111.7 [110.2–135.7]	110.5 [99.8–124.9]
		Week 12	115.5 [94.8–141.9]	123.3 [99.5–149.6]
	Total cholesterol (mg/dL)	Baseline	207.0 [183.0–220.0]	207.3 [188.6–229.1]
		Week 12	181.3 [166.8–222.8]	205.9 [179.8–224.3]
Intervention	Goat / cow milk allocation	Group Distribution	2 / 1	7 / 9

Note: Continuous variables are presented as median [interquartile range]. These data are descriptive and exploratory because only three participants had *Blastocystis* PCR detection at baseline and Week 12. The table is not intended to assess group equivalence, confounding, clinical differences, or metabolic associations.

Supplementary Table S2. Descriptive Anthropometric and HOMA-IR Measurements for Participants with *Blastocystis* Detected at Baseline and Week 12

Participant	Intervention arm	Subtype	Time point	*Cryptosporidium* spp. and *Giardia duodenalis* PCR result	Body fat, %	BMI, kg/m²	HOMA-IR
G10	Goat milk	ST4	Baseline	Negative	28.7	24.53	1.10
G10	Goat milk	ST4	Week 12	Negative	28.6	24.24	0.50
G16	Goat milk	ST2	Baseline	Negative	14.5	22.41	0.50
G16	Goat milk	ST2	Week 12	Negative	13.1	21.57	0.40
C23	Cow milk	ST2	Baseline	Negative	27.5	22.93	1.10
C23	Cow milk	ST2	Week 12	Negative	23.5	21.46	2.10

Note: Data are presented descriptively for transparency. *Blastocystis* was detected by PCR at baseline and Week 12 in these three participants. Individual measurements were not interpreted as evidence of treatment effects, metabolic benefit, or subtype-specific effects. All 38 samples were negative for *Cryptosporidium* spp. and *Giardia duodenalis* by the specified nested-PCR assays.

Supplementary Table S3. Processed Sequencing Reads Before and After Rarefaction

Sample ID	Processed sequencing reads before rarefaction	Sequencing reads retained after rarefaction
G2a	46,517	22,455
G2b	45,921	22,455
C3a	22,455	22,455
C3b	38,026	22,455
G4a	47,679	22,455

Sample ID	Processed sequencing reads before rarefaction	Sequencing reads retained after rarefaction
G4b	33,485	22,455
C5a	36,513	22,455
C5b	49,777	22,455
G6a	41,491	22,455
G6b	45,526	22,455
C7a	49,925	22,455
C7b	37,878	22,455
G8a	42,500	22,455
G8b	43,175	22,455
C9a	44,759	22,455
C9b	48,547	22,455
G10a	53,679	22,455
G10b	33,509	22,455
C11a	49,382	22,455
C11b	44,111	22,455
C13a	48,704	22,455
C13b	42,068	22,455

Sample ID	Processed sequencing reads before rarefaction	Sequencing reads retained after rarefaction
G14a	38,090	22,455
G14b	56,490	22,455
C15a	37,114	22,455
C15b	39,452	22,455
G16a	40,112	22,455
G16b	39,416	22,455
C17a	30,323	22,455
C17b	31,460	22,455
G20a	56,293	22,455
G20b	50,949	22,455
C21a	33,860	22,455
C21b	32,435	22,455
G22a	40,376	22,455
G22b	38,528	22,455
C23a	46,036	22,455
C23b	39,135	22,455

Note: Processed sequencing-read counts were obtained from the non-rarefied Novogene ASV feature table. The rarefaction depth of 22,455 reads per sample was selected because it represented the minimum processed sequencing depth among the 38 stool samples and retained all samples for alpha-diversity analysis. C3a had 22,455 processed sequencing

reads before rarefaction; all remaining samples were randomly subsampled to 22,455 reads per sample. Sample IDs ending in "a" indicate baseline and those ending in "b" indicate Week 12.

Supplementary Table S4. PERMDISP Analysis of Bray-Curtis Dissimilarities by *Blastocystis* Detection Status

Time point	Mean distance to centroid, detected, n = 3	Mean distance to centroid, not detected, n = 16	pseudo-F	Permutation p-value
Baseline	0.366	0.377	0.026	0.8531
Week 12	0.423	0.502	2.274	0.1517

Note: PERMDISP was based on Bray-Curtis dissimilarities calculated from genus-level relative abundances derived from non-rarefied processed 16S rRNA gene count data. Permutation tests used 9999 permutations. Non-significant results indicate that differential multivariate dispersion was not detected under the specified analyses; they do not demonstrate equal dispersion between groups.

Supplementary Table S5. Exploratory Relative-Abundance Comparisons for Eight Selected Bacterial Genera According to *Blastocystis* PCR-Detection Status

Time point	Genus	*Blastocystis* detected, n = 3, median [IQR] %	*Blastocystis* not detected n = 16, median [IQR] %	Unadjusted p-value	FDR q-value
Baseline	*Akkermansia*	1.00 [0.50–2.08]	0.01 [0.00–0.32]	0.5593	0.7458
	Alistipes	1.21 [0.61–2.10]	0.00 [0.00–0.08]	0.0475	0.3385
	Bifidobacterium	5.64 [4.14–9.06]	17.10 [10.03–32.22]	0.1713	0.4568
	Escherichia-Shigella	0.00 [0.00–0.18]	0.00 [0.00–0.00]	0.7926	0.7926
	Faecalibacterium	13.03 [6.85–16.92]	0.66 [0.12–2.98]	0.0846	0.3385
	Methanobrevibacter	0.00 [0.00–1.55]	0.00 [0.00–6.94]	0.7926	0.7926

Time point	Genus	*Blastocystis* detected, *n* = 3, median [IQR] %	*Blastocystis* not detected *n* = 16, median [IQR] %	Unadjusted *p*-value	FDR *q*-value
	Oscillospira	0.00 [0.00–0.01]	0.00 [0.00–0.00]	0.4211	0.6737
	Prevotella	0.00 [0.00–0.05]	0.00 [0.00–0.00]	0.4211	0.6737
Week 12	*Akkermansia*	0.22 [0.11–2.21]	0.08 [0.01–0.17]	0.5593	0.7458
	Alistipes	1.12 [0.59–1.79]	0.05 [0.00–0.16]	0.0846	0.6770
	Bifidobacterium	5.26 [3.11–10.87]	11.80 [6.03–25.66]	0.3034	0.7458
	Escherichia-Shigella	0.03 [0.01–0.61]	0.19 [0.02–1.53]	0.7926	0.9058
	Faecalibacterium	0.83 [0.43–7.71]	0.12 [0.01–1.80]	0.4871	0.7458
	Methanobrevibacter	0.00 [0.00–6.92]	0.00 [0.00–2.06]	1.0000	1.0000
	Oscillospira	0.00 [0.00–0.01]	0.00 [0.00–0.00]	0.5593	0.7458
	Prevotella	0.00 [0.00–0.00]	0.00 [0.00–0.00]	0.5593	0.7458

Note: Relative abundances were obtained from the Novogene genus-level relative-abundance table derived from ASV data rarefied to 22,455 reads per sample. Two-sided Mann-Whitney U tests were conducted separately at baseline and Week 12. Benjamini-Hochberg adjustment was applied within each time point across the eight selected genera. No comparison met the applied false-discovery-rate-adjusted threshold of $q < 0.05$. Unadjusted *p*-values are reported for transparency and were not interpreted as confirmatory evidence of between-group differences. Zero values indicate that no reads assigned to the taxon were observed after processing and rarefaction; they do not demonstrate biological absence.

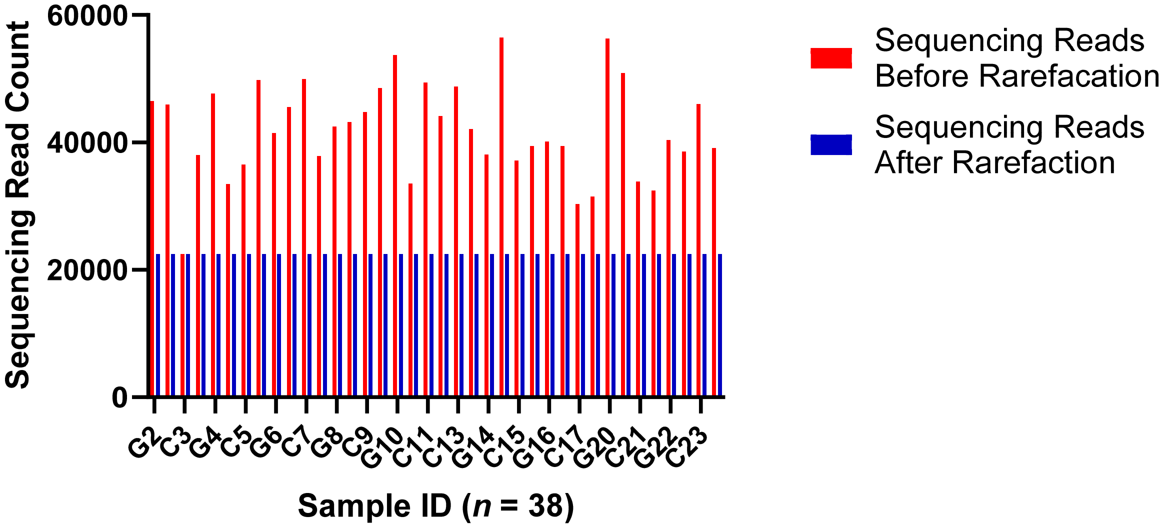

Supplementary Figure S1. Processed sequencing depth across the 38 stool samples. Bars represent processed sequencing reads before rarefaction. The horizontal dashed line indicates the selected rarefaction depth of 22,455 reads per sample, which retained all samples for alpha-diversity analysis. Sample IDs contain baseline and Week 12.
